# Molecular Biomarkers in Borderline Ovarian Tumors: Towards Personalized Treatment and Prognostic Assessment

**DOI:** 10.3390/cancers17030545

**Published:** 2025-02-06

**Authors:** Stefania Drymiotou, Efthymia Theodorou, Kathrine Sofia Rallis, Marios Nicolaides, Michail Sideris

**Affiliations:** 1 Barts and the London School of Medicine and Dentistry, Queen Mary University of London, London E1 2AD, UK; stephania.drymiotou1@nhs.net (S.D.); efthymia.theodorou@nhs.net (E.T.); ha16849@qmul.ac.uk (K.S.R.); 2 Guy’s and St Thomas’ NHS Foundation Trust, Westminster Bridge Road, London SE1 7EH, UK; marios.nicolaides@nhs.net; 3 Wolfson Institute of Population Health, Queen Mary University of London, Charterhouse Square Campus, Barbican, London EC1M 6BQ, UK

**Keywords:** borderline ovarian tumours (BOTs), molecular biomarkers, KRAS, BRAF, Ca125, calprotectin, microsatellite instability (MSI), p16ink4a

## Abstract

Borderline Ovarian Tumours (BOTs) are a heterogenous group of ovarian neoplasms which have increased mitotic activity but lack stromal invasion. We performed a narrative review of the literature, aiming to identify prognostic molecular biomarkers that can potentially be used for treatment personalisation. We identified and discussed BRAF/KRAS, Cancer Antigen 125 (Ca 125), Calprotectin, p16ink4a, and Microsatellite instability (MSI) as the most studied biomarkers related to BOTs. Overall, BRAF and KRAS mutations are associated with earlier-stage and favourable prognosis; KRASmt may indicate extraovarian disease in serous BOT (sBOT). Ca125, the only currently clinically used biomarker, can be assessed pre-operatively and has an established role in post-operative surveillance, especially when it is raised pre-operatively or a high potential for malignant transformation is suspected post-operatively. p16ink4a expression trends could also indicate the malignant transformation of the tumour. Calprotectin has an inferior specificity to Ca125 and is not yet established as a biomarker, whilst there is very limited evidence available for MSI. As new evidence is coming along with artificial intelligence platforms, these biomarkers can be integrated and used towards the development of a precision model for treatment stratification and counselling in women diagnosed with BOTs.

## Simple Summary

Borderline Ovarian Tumors (BOTs) are a unique group of ovarian growths that fall between benign and malignant categories. These tumours primarily affect younger women, often raising concerns about preserving fertility during treatment. Currently, doctors rely on surgical findings and tumour characteristics to predict outcomes and guide treatment decisions. However, this approach may not always provide a complete picture. Our review explores the potential of using molecular markers, such as BRAF, KRAS, CA125, and others, to better understand and manage BOTs. By identifying these biomarkers, we aim to develop a more personalized approach to treatment, potentially allowing for less aggressive interventions in low-risk cases while ensuring appropriate care for those at higher risk of recurrence or progression. This research could lead to improved decision-making and outcomes for women diagnosed with BOTs.

## 1. Introduction

As of 1961, the International Federation of Gynaecology and Obstetrics (FIGO) has recognized borderline ovarian tumours (BOTs), or low malignant potential tumours, as a distinct entity of ovarian neoplasms which typically lack stromal invasion, which is a hallmark of invasive cancers [1,2]. This has also been adopted by the World Health Organisation (WHO) in 1973 [1]. Histologically, BOTs show nuclear atypia, significant mitotic activity, and have hierarchically branched papillae [3,4]. Based on their histology, BOTs are further divided to serous, which are the main subtype (approximately 53–65% of BOTs), followed by mucinous, which comprise 32–42% of the total. The rest, less than 5%, constitute endometrial tumours, clear-cell tumours, and transitional-cell or Brenner’s tumours [2,5].

BOTs account for approximately 15–20% of all epithelial ovarian tumours [6], with an incidence of 1.8–4.8 per 100,000 women per year [2]. In contrast to carcinomas, BOTs affect women of a younger age group and have a better overall survival. Ten-year overall survival for any stage is quoted as 95% [6]. Since 27–54% of women that present with BOTs are younger than 40 years, many of them would wish to preserve fertility. Currently, the gold standard management remains surgical staging [7]. This includes unilateral/bilateral salpingo-oophorectomy, omentectomy with or without hysterectomy, and peritoneal biopsies, depending on fertility aspirations. Complete macroscopic tumour resection should be the aim of all surgery for BOTs, with adequate surgical staging, especially in apparent stage 1 disease, including peritoneal biopsies, omentectomy, and cytology. However, since women presenting with BOTs tend to be relatively young, fertility-sparing surgery can be offered and does not preclude adequate peritoneal staging along with unilateral salpingo-oophorectomy (USO), with or without contralateral ovarian cystectomy, preserving the non-affected ovary and the uterus [5].

Currently, treatment stratification and prediction of the overall prognosis, including cancer progression or possible short- and long-term recurrence, are based on the FIGO stage, the presence of peritoneal implants, the micropapillary pattern, and microinvasion. Additionally, incomplete staging, residual tumours, and fertility-sparing surgery are independent prognostic factors contributing to higher recurrence rates [8,9].

For instance, in patients with peritoneal implants, those with invasive implants appear to have a higher relapse rate (>50%) and worse prognosis than those with non-invasive implants (20–50%) [10]. Several studies support the use of additional factors as predictive or prognostic; these include incomplete surgical staging, residual disease, fertility-sparing surgery, preoperative serum CA 125 levels, bilateral ovarian involvement and age. However, there is still no robust conclusion as to whether these factors have an established role in the prediction of survival to further stratify the treatment approach. As a result, treatment approaches for BOTs can still face several dilemmas and remain an uncertain field for clinicians, who seek a multidisciplinary team-based approach to optimize patients’ survival while respecting their future fertility wishes.

On this basis, we aimed to identify and discuss serum or tissue biomarkers which could potentially be used as a useful clinical decision adjuncts in prognostic and treatment stratification. This would allow the development of a more personalized treatment approach for BOTs.

## 2. Materials and Methods

We conducted a narrative review of the literature to identify studies discussing the role of prognostic and predictive molecular biomarkers in managing BOTs. Two independent reviewers (ET/MN) performed an initial search on Pubmed, which yielded studies on various biomarkers. Based on this, we refined our focus to a set of ‘globally accepted’ biomarkers commonly used in managing gynecological cancers. We defined ‘globally accepted’ as biomarkers that were the primary focus of most original BOT studies, a criterion confirmed during a consensus meeting with the senior author (MS). Hence, two independent pairs of reviewers (SD/ET, KR/MN) performed a second structured search based on the shortlisted panel of biomarkers: this included the V-raf murine sarcoma viral oncogene homolog B (BRAF), Cancer Antigen 125 (Ca 125), calprotectin, p16ink4a, Kirstin rat sarcoma oncogene homolog (KRAS), and microsatellite instability (MSI). A summary is provided in Table 1.

## 3. Results

### 3.1. V-Raf Murine Sarcoma Viral Oncogene Homolog B (BRAF)

BRAF encodes a serine–threonine protein kinase in the RAS-RAF-mitogen/extracellular signal-regulated kinase (MEK), extracellular signal-regulated kinase (ERK), and mitogen-activated protein kinase (MAPK) pathway, which acts as a downstream effector of the Kirsten rat sarcoma viral oncogene homolog (KRAS) [11,12]. Oncogenic forms of BRAF have been identified in many tumours, including malignant melanomas, colorectal cancer, lung, papillary thyroid, and ovarian neoplasms [13,14,15].

Activating mutations in the BRAF proto-oncogene lead to activation of the MAPK signalling pathway, which is involved in the growth and differentiation of tissues [16]. The most commonly seen BRAF activating mutation occurs within or immediately adjacent to the activation segment in exon 15. Specifically, this involves a single substitution of A for a T at nucleotide position 1796, resulting in the exchange of a valine for glutamate, at position 600 (V600E) [11,12]. This substitution stabilizes the active conformation of the kinase, resulting in BRAF being continuously active, independent of upstream signalling, leading to malignant cell transformation [16,17,18].

BRAF mutations in serous BOTs have been extensively described in the literature with an incidence of 23–48% [19,20]. The role of BRAF mutations in mucinous BOTs is not as well studied. Anglesio et al. (2008) and Hunter et al. (2012) have described the prevalence of BRAF mutations in this BOT histotype as 5 and 14%, respectively, whereas Ohnishi et al. (2020) found the BRAFV600E mutation to occur in 40% of the samples studied [21,22,23]. BRAF mutations can occur in low-grade serous ovarian carcinomas (LGSCs) and their presence is associated with an earlier stage of disease at presentation, better prognosis, and they are less frequently seen in advanced or recurrent LGSC [20,24,25,26,27]. The incidence of BRAF mutations in LGSCs is 0–33% [19]. It has been suggested that these mutations may have a protective role against malignant transformation of BOTs as they more stable mutations, can enhance the expression of tumour suppressor genes, as well as induce cellular senescence [28,29,30,31,32]. Therefore, BOTs lacking BRAF mutations are more likely to progress to aggressive LGSCs or recur [19,26,30].

Molecular profiling of BOTs and testing for BRAF mutations serve as valuable tools, particularly when planning fertility-sparing treatments for women who wish to continue having children. BRAF mutations are a favourable prognostic marker due to their strong association with a low recurrence risk and indolent behaviour which may offer clinicians greater confidence in adopting conservative management strategies. In rare cases where patients with BRAF mutations experience malignant transformation or recurrence and have depleted treatment options, they may be eligible for treatment with BRAF inhibitors or combination regimens involving BRAF and MEK inhibitors, although research in this area is still limited [33,34,35,36,37].

Ho et al. (2004) analyzed a small sample of eight serous BOTs associated with serous cystadenomas to explore the role of BRAF and KRAS mutations during the early stages of ovarian serous development [38]. The study demonstrated that mutations in either BRAF or KRAS were found in 88% of serous BOTs, with these mutations being mutually exclusive. This highlights their early and distinct roles in ovarian tumourigenesis. The adjacent cystadenomas harboured identical mutations in most cases, strongly suggesting that these benign lesions may serve as precursors to BOTs. Identifying BRAF mutations during molecular profiling does not exclude the diagnosis of benign serous cystadenomas. However, this information can be pivotal in clinical contexts where distinguishing between cystadenomas and BOTs is ambiguous.

### 3.2. Cancer Antigen 125—CA 125

CA 125 is the most commonly used tumour marker in ovarian cancer surveillance and is frequently contemplated to be the ‘gold standard’ in clinical practise. CA 125 is a high-molecular-weight glycoprotein with two major antigenic domains and is expressed by fetal amniotic and coelomic epithelium and in adult tissues derived from the coelomic and Mullerian epithelia. It is raised in approximately 90% of patients with advanced epithelial ovarian cancer and since its development it has become embedded in the management of ovarian cancer [39]. Nevertheless, its value as an ovarian cancer diagnostic marker is limited by the fact that it is non-specific and can be found in high levels in several circumstances [40].

The association of Ca125 with BOTs has been widely studied over the years. Studies report high serum Ca125 in 24–61% patients with BOTs [41] and conclude that women with BOT have a lower median serum CA125 compared to women with invasive ovarian cancers [42], but higher levels than healthy controls [43]. Specifically, Lehard et al. report that the median level of Ca125 in women with BOTs was 34.7 U/mL (range 18.1–385.0 U/mL) compared to 13.5 U/mL (range 4.0–49.7 U/mL), in the control group and 401.5 U/mL (range 12.5–35,813 U/mL) in women with ovarian cancer [43]. The wide range in all groups, however, means that there can be overlap between patients who have early-stage ovarian cancer and patients with BOTs and thus it is of limited benefit in distinguishing pre-operatively between BOTs and invasive ovarian cancer. Stage I BOT exhibits a trend of lower Ca125 presence, when compared to stages II, III and IV [44,45,46,47].

Ca125 has a role in post-op surveillance, especially in the case where this is found to have increased pre-operatively. It is also one of the most sensitive biomarkers in the advanced stage at presentation. In such cases, follow-up surveillance is predominantly focused on capturing malignant transformation as early as possible. A rise in Ca125 can be regarded as the earliest sign of malignant transformation to LGSOC [6].

### 3.3. Calprotectin

Calprotectin, a member of the S-100 protein family, is a calcium- and zinc-binding protein primarily found in the cytosol of human neutrophil granulocytes and monocytes [48]. It serves as an inflammation marker, released from neutrophils upon activation, and exhibits antimicrobial properties, along with pro-inflammatory cytokine functions and chemotactic factor activity [48].

Elevated calprotectin levels are indicative of poor cell differentiation, suggesting its involvement in tumour development. It has been overexpressed in various malignancies, including hepatocellular carcinomas, pulmonary adenocarcinomas, invasive ductal carcinomas of the breast, squamous cell carcinomas of the lung, colorectal carcinomas, and primary tumours in prostate cancer [49].

Several studies have identified various biomarkers in ovarian cystic fluid, including calprotectin. For instance, Ott et al. reported elevated calprotectin levels in both ovarian cystic fluid and serum from women with ovarian neoplasia (n = 11) compared to those with benign ovarian cysts (n = 11) [50]. Ødegaard et al. conducted multiple studies that demonstrated women with epithelial invasive ovarian cancer had higher preoperative levels of calprotectin than those with borderline ovarian tumours (BOTs) or concurrent BOT and ovarian cancer. Their findings indicated that median plasma calprotectin concentrations were similar in women with BOTs and those with benign tumours [48,51]. However, these results are limited by the fact that calprotectin did not exhibit significant prognostic value. They suggested that calprotectin may have superior diagnostic capacity for differentiating invasive cancer compared to other novel circulating biomarkers like macrophage-inhibiting factor. Additionally, their study noted that calprotectin levels decreased post-chemotherapy (either platinum or paclitaxel), raising interest in its potential as a predictive biomarker [48,52].

In conclusion, while the specificity of calprotectin is considered inferior to CA-125—given that many women with BOTs present with plasma calprotectin concentrations above clinically relevant thresholds—its elevated levels in ovarian carcinoma could indicate advanced stages of the carcinogenesis pathway. Thus, while calprotectin may cautiously serve as a biomarker for invasive disease, its overall prognostic value remains uncertain for clinical practise [49,53].

### 3.4. P16INK4a

The principal member of the INK4 family of cyclin-dependent kinase (CDK) inhibitors, p16INK4a, is encoded by the CDKN2A gene located on the short arm of chromosome 9 [54,55]. It is a tumour-suppressor protein [12,56] involved in cell cycle regulation by inhibiting CDK4 and CDK6, which is necessary for the subsequent phosphorylation of the retinoblastoma protein (pRb) [12,57,58,59]. This inhibits cell cycle progression from the G1 to S phase, suppressing cell proliferation. p16INK4a serves as a marker of senescence, with its expression increasing both during ageing and in response to stress stimuli [57,60,61]. Being a tumour suppressor, the main role of p16INK4a is to prevent tumour formation and is inactivated in approximately 50% of human malignancies including pancreatic, bowel, breast, and lung cancers [55,62,63,64]. This inactivation often occurs through different mechanisms that include gene deletion, promoter hypermethylation leading to transcriptional silencing, and point mutations that interfere with p16 function [65,66,67,68,69]. HPV-related cancers, however, such as cervical and oropharyngeal malignancies, exhibit p16INK4a overexpression because of how HPV interferes with the cell cycle [70,71,72]. The viral E7 oncoprotein binds to and inactivates pRb, disrupting cell cycle regulation. Normally, pRb suppresses p16INK4a expression as part of its regulatory role and once pRb is inactivated, the feedback loop is disrupted and p16INK4a is overexpressed.

The value of p16INK4a expression (either absence or overexpression) as a prognostic marker in ovarian cancer is histotype-dependent [73]. Rambau et al. (2018) [73] demonstrated p16INK4a overexpression in HGSC but this was not associated with shorter overall survival in this histotype. In endometriosis-associated, clear cell, and endometrioid carcinomas, however, p16INK4a was overexpressed and associated with shorter overall survival. The authors also reported the absence of p16INK4a expression in LGSC, which was associated with shorter survival. A meta-analysis conducted by Ruan et al. (2018) concluded that p16INK4a promoter methylation was increased in ovarian carcinomas compared to BOTs and benign tissues [74]. In addition, p16INK4a promoter methylation was associated with a shorter progression-free survival. Xia et al. (2019), in their meta-analysis, demonstrated that CDKN2A, CDKN2B, and CDH13 promoter methylation was higher in ovarian carcinomas compared to normal tissues [75]. CDKN2A promoter methylation also correlated with poorer progression-free survival but not overall survival [75]. A meta-analysis by Jiang et al. (2017) concluded that p16INK4a promoter methylation has limited value in differentiating malignant from benign ovarian tumours [76]. However, this study only used data from the previous literature, unlike Ruan et al. (2018) and Xia et al. (2019), who also included data from the Cancer Genome Atlas Program (TCGA) and, therefore, the Jiang et al. (2017) meta-analysis had limited clinical relevance. Unlike Ruan et al. (2018), which quantitatively assessed the extent of p16INK4a methylation, potentially identifying thresholds or patterns relevant to ovarian cancer, Jiang et al. (2017) only tested the association [74,75,76] between p16INK4a methylation and ovarian carcinoma.

Despite the extensive evidence in the relationship of p16INK4a and ovarian cancer, more limited is the evidence for the association between p16INK4A and borderline ovarian tumours. Cabral et al. (2016) demonstrated increased p16INK4A expression frequency from benign (45.6%) to borderline (75%) to malignant (94.6%) (*p* = 0.000) tumours, demonstrating that p16INK4a expression might show progressive upregulation or differential regulation as tumours advance from benign to malignant stages [77]. In fact, this study primarily focused on p14, another product of the CDKN2A locus, and demonstrated the differential expression of this protein in benign, borderline, and malignant epithelial ovarian tumours. The expression of p14 is low or absent in benign cases, intermediate in BOTs, and high or aberrant in malignant carcinomas, highlighting that p14 expression patterns can be used as a potential diagnostic biomarker. In addition, high p14 expression in malignant tumours may support its role as a marker of tumour aggressiveness or malignancy [77]. Yoon et al. (2016) have reported that stromal p16INK4a expression is significantly increased in BOTs compared to benign lesions (*p* < 0.001) and in malignant tumours compared to BOTs (*p* < 0.001) [78]. This study suggests that p16INK4a is not only altered in epithelial tumour cells but also in the surrounding stroma, reflecting its broader role in the tumour microenvironment (TME). Elevated stromal p16INK4a expression may correlate with malignancy and tumour aggressiveness, making it a potential marker which can be used as an adjunct to epithelial p16INK4a expression for differentiating malignant ovarian neoplasms from benign or borderline cases [78].

In addition, p16INK4a expression may serve as a potential biomarker to help stratify patients for treatment after surgery. For example, Wang et al. (2018) demonstrated that p16INK4a and Trp53 tumour status could serve as biomarkers to predict the efficacy of Olaparib in inducing senescence in ovarian cancer patients. Tumours retaining either p16INK4a and Trp53 activity were more likely to respond to Olaparib [79]. Tu et al. (2023) investigated the role of p16INK4a as a biomarker for predicting outcomes and guiding therapies, in the context of immune checkpoint inhibitors (ICIs) [80]. High p16INK4a expression is associated with increased immune cell infiltration in the TME, making tumours more responsive to ICIs. Ovarian cancer is known for its immunosuppressive TME, yet some ovarian tumours, particularly those with a high mutational burden or DNA repair defects, may respond to ICIs [81,82,83]. If p16INK4a expression correlates with increased immune cell infiltration in ovarian cancer, it could serve as a biomarker for identifying patients more likely to benefit from ICIs. Since p16INK4a expression is a marker of cellular senescence, combining ICIs with senescence-modulating therapies could be another therapeutic strategy in women with tumours exhibiting high p16INK4a expression and enhanced immune cell infiltration [84].

These findings suggest that both epithelial and stromal p16INK4A expression could be used as a potential borderline ovarian tumour biomarker in combination with other biomarkers. High expression trends may represent a particular BOT cohort that could have a higher potential to progress from borderline to invasive disease, and could therefore guide both surgical and pharmacological management, especially in cases where fertility-sparing surgery is considered.

### 3.5. Ki-Ras2 Kirsten Rat Sarcoma Viral Oncogene Homolog—KRAS

KRAS is a proto-oncogene located on chromosome 12p12 that encodes a 21-kD protein (p21RAS) involved in extracellular signalling [12]. Mutations in KRAS activate downstream signalling in the MAP-kinase pathway, which regulates cellular proliferation and differentiation, and can also downregulate the PI3K-AKT pathway, which is involved in cell survival and growth. Overall, 90% of KRAS mutations refer to an alteration of G>A, either in codons 12 or 13 of exon 2 [11,12,85]. Most KRAS mutations contribute to constitutive activation of this signal transduction pathway and the resultant uncontrolled proliferation and differentiation of cells and hence KRAS is involved in a plethora of human carcinogenesis pathways [11,12,86].

There has been sufficient evidence to support that low-grade serous carcinoma (LGSOC) is distinct from high-grade serous (HGSOC) and is associated with BRAF and KRAS mutations. Oncogenic activation of the MAPK pathway by KRAS mutations is a common feature of sBOTs [87]. BOTs can be precursors of LGSOC [88] and hence the role of KRAS in this progression remains a pivotal question. Generally, KRAS and BRAF mutations are mutually exclusive [88,89]. KRAS mutations in codons 12 and 13 or codon 599 of BRAF occur in approximately 2/3 of sBOT cohorts and LGSOC [90]. Although the role of KRAS has been predominantly studied in the concept of sBOT and extraovarian implants, there is some evidence that such mutations can be found in mucinous BOTs as well, with studies reporting presence as high as 60% [90,91].

There is emerging evidence that KRAS mutation status may be a prognostic indicator for extraovarian implants [92]. In a study published at Yale University, KRAS mutation was present in 12/20 invasive implants (60%) and 3/22 non-invasive implants (14%), leading to the conclusion that KRASmt may be a feature of progression to invasive disease. For women in whom extraovarian disease is identified, the aim of the follow up is to identify progression to invasive disease early. On these grounds, although evidence still slim, KRAS may be the right biomarker to indicate such progression. The same team claimed that KRAS mutations indicate a more aggressive tumour behaviour with higher recurrence and worse disease-specific survival. Another study by Mc Henry et al. [87] indicated a similar trend of KRAS mutations in LGSOC (invasive implants) vs. noninvasive implants. Similar to the Yale team, McHenry claimed that KRAS mutation status in primary sBOTs indicates worse disease-free survival, independent of histological subtype or tumour stage or the presence of extraovarian disease, concluding that sBOT disease with a KRAS mutant pattern indicates a perhaps more aggressive biological entity with a higher likelihood of recurrence [87]. A review article from a Brazilian team [93] supports KRAS mutation status as an indicator for higher recurrence rates and more progression to LGSOC.

Contrary to the previous studies, a German group [94] states that although KRAS and/or BRAF mutations are a feature of BOTs, their presence does not necessarily indicate invasive disease. Similarly, a team from Memorial Sloan Kettering (MSK) [89] did not associate KRAS mutation status with invasive disease or more aggressive tumour behaviour or advanced stage of disease. In the same study, the presence of a BRAF mutation was a favourable prognostic marker. A Polish team of researchers also did not support the mutational status of KRAS as a prognostic biomarker [95]. Those discordances can be attributed to the small number of the studies. Equally, BOTs are a highly heterogenous group of tumours where “one size does not fit all”. As a large cascade of genomic events contribute to a more aggressive pattern of BOTs with extraovarian spread and potentially malignant transformation [91], KRAS mutation alone may not be sufficient to explain this on its own but rather as part of a bigger process [96,97].

### 3.6. Microsatellite Instability (MSI)

Microsatellites (MS) are short, repetitive DNA sequences which are prone to frame-shift type mutations as well as base-pair substitutions during replication [12]. MS are encoding DNA repair genes which are essential for the replication of the DNA during cell proliferation [12]. Insertion or deletion of repeating units during DNA replication can cause Microsatellite instability (MSI). Although MSI has an established role for a variety of cancers, there is limited evidence on its role in the BOT pathway.

Some very early efforts from the US pointed to the presence of MSI in 2/5 sBOT specimens, raising some novel research hypotheses [98]. A Spanish group indicated that BAT26/D2S123 instability was detected in BOTs but those findings were inconclusive as regards any prognostic significance [99], which underlines the unclear role of MSI in BOTs. Therefore, more prospective evidence is required to support any role of MSI in the ΒOΤ pathway.

## 4. Discussion

### 4.1. Summary of Our Findings

This review highlights key molecular biomarkers associated with BOTs, focusing on their potential prognostic and predictive utility. Among the discussed markers, BRAF and KRAS mutations emerge as significant due to their association with favourable prognosis and their roles in the early stages of tumorigenesis. These mutations, often mutually exclusive, are prevalent in serous BOTs and low-grade serous carcinomas (LGSCs). BRAF mutations, particularly V600E, are linked to indolent disease behaviour and a reduced risk of progression to invasive carcinomas. KRAS mutations, albeit less definitively prognostic, demonstrate potential relevance in predicting the recurrence and spread of extraovarian disease. Evidence concludes that BRAF/KRAS mutations can also be present in benign ovarian tumours as well as in serous and mucinous BOTs; this supports the hypothesis that such mutations are early features in the low-grade serous carcinoma pathway, potentially representing more favourable prognosis in that setting [38].

This study also evaluates stromal and epithelial p16INK4a expression, noting its gradual upregulation from benign lesions to malignant carcinomas, which could indicate malignant transformation. However, evidence for p16INK4a’s role in BOTs remains limited. Furthermore, this review confirms the limited specificity of serum biomarkers like Ca125 and calprotectin for BOTs, though Ca125 retains utility in post-operative surveillance, particularly for advanced disease. Ca125 is the only biomarker used in clinical practise (sBOT), and has potential to identify the malignant transformation of sBOTs, especially in non-invasive implants. Microsatellite instability (MSI), despite its established role in other cancers, lacks robust evidence supporting its prognostic or predictive value in BOTs.

### 4.2. Clinical Implications

The prognostic value of BRAF and KRAS mutations underscores their utility in post-operative treatment planning, particularly for fertility-preserving surgery in younger women. Identifying these mutations may inform decisions regarding completion surgery and guide clinicians in balancing oncologic safety with fertility preservation. However, as these mutations can only be assessed post-operatively, their role is limited to adjunctive decision-making rather than pre-operative stratification. Further to this, although evidence is promising, it is yet unclear how KRAS/BRAF status should be interpreted. For instance, whether KRASmt non-invasive implants have the potential to transform into LGSOC; current evidence is very slim to suggest such conclusions.

Ca125, despite its limited specificity, remains the most practical pre-operative biomarker due to its accessibility and established role in ovarian cancer management. Elevated pre-operative Ca125 levels warrant closer monitoring for potential malignant transformation or recurrence. Certainly, although not discussed in this review, Ca19-9 also holds a place in post-operative follow up of mBOTs. On the other hand, calprotectin’s lower specificity and lack of prognostic value restrict its utility in clinical practise.

The potential of p16INK4a as a biomarker for early-stage malignancy warrants further exploration, particularly in its dual expression in stromal and epithelial compartments. Its upregulation may offer additional insights into tumour progression, aiding in risk stratification and treatment planning. MSI’s minimal role in BOTs suggests that research efforts should focus elsewhere. Clinical implication of each biomarker is annotate on Table 2.

### 4.3. Limitations

This review’s narrative approach and the heterogeneity of BOTs pose challenges in synthesizing consistent conclusions. Many studies combine BOTs with invasive ovarian cancers, complicating the extrapolation of biomarker-specific findings. Furthermore, the variability in genetic mutations, even within the same biomarker (e.g., BRAF codon 600 vs. 599 mutations), introduces additional complexity. Future collaborative efforts focusing on individual biomarkers or employing meta-analytic (evidence synthesis) techniques may yield more definitive insights.

## 5. Conclusions

Given the complexity of molecular oncology, a single-biomarker approach has proven insufficient for comprehensive treatment stratification. Instead, integrating a panel of biomarkers with established histopathological features could offer a more nuanced model for predicting prognosis and guiding treatment in BOTs. Emerging research on extracellular miRNA and proteomics offers [100,101] promising avenues for identifying minimally invasive biomarkers that could complement existing strategies.

Additionally, the application of artificial intelligence (AI) [102] in synthesizing large datasets and integrating biomarker profiles with clinical parameters may revolutionize BOT management. AI-driven precision medicine could facilitate individualized treatment planning, optimizing oncologic outcomes while addressing fertility preservation concerns. This aligns with the broader vision of personalized and precision oncology.

## Figures and Tables

**Table 1:** Summary of discussed biomarkers.

Biomarker	Summary
**KRAS**	Oncogene involved in RAS-RAF-MEK-ERK pathway. Mutations associated with earlier stage BOTs and favourable prognosis. In serous BOTs, KRAS mutations may indicate extraovarian disease. Mutually exclusive with BRAF mutations, playing a role in early ovarian tumourigenesis.
**BRAF**	Serine–threonine protein kinase in RAS-RAF-MEK-ERK pathway. Mutations (23–48% in serous BOTs) associated with earlier stage, better prognosis, and lower recurrence risk. May have a protective role against malignant transformation. Potentially useful following further research on planning fertility-sparing treatments and potential targeted therapies.
**p16ink4a**	Limited information provided in search results. Expression trends could indicate malignant transformation of tumour. Further research needed to establish its role as biomarker in BOTs.
**CA125**	Most commonly used tumour marker in ovarian cancer. Elevated in 24–61% of BOT patients. Lower median levels compared to invasive ovarian cancers, but higher than healthy controls. Useful in post-operative surveillance, especially when pre-operatively elevated. May indicate malignant transformation to LGSOC when rising.
**Calprotectin**	Calcium- and zinc-binding protein, inflammation marker. Overexpressed in various malignancies. Identified in ovarian cystic fluid. Shows inferior specificity compared to CA125 for BOTs. Not yet established as reliable biomarker for BOTs. Further research needed to determine its clinical utility.
**MSI**	Very limited evidence available in search results. Microsatellite instability is mentioned as one of studied biomarkers related to BOTs, but no specific information is provided about its role or significance in BOT diagnosis or prognosis.

**Table 2:** Clinical implication of each biomarker.

Biomarker	Clinical Implications
KRAS	- May help identify BOTs with a higher risk of malignant transformation, but evidence is inconsistent. - Could assist in stratifying high-risk patients for closer follow-up. - Potential therapeutic target in advanced or recurrent cases.
BRAF	- May support conservative or fertility-sparing treatment decisions in young patients. - Considered a favourable prognostic marker, associated with lower recurrence risk. - Potential candidate for targeted therapy (BRAF/MEK inhibitors) in rare cases of malignant transformation.
p16INK4a	- May serve as a prognostic marker in combination with other biomarkers. - Could help identify BOTs at risk of progression, guiding surgical and follow-up strategies.
Ca125	- Useful in postoperative surveillance, particularly when preoperative levels are elevated. - Can help monitor malignant transformation but lacks specificity to distinguish BOTs from invasive cancers.
calprotectin	- Not yet established as a reliable biomarker for BOTs. - May indicate progression in advanced-stage disease, but further studies are needed.
MSI	- Currently not a reliable biomarker for BOTs. - Further research needed to determine any potential diagnostic or prognostic value.
